# Military Veterans and Their PTSD Service Dogs: Associations Between Training Methods, PTSD Severity, Dog Behavior, and the Human-Animal Bond

**DOI:** 10.3389/fvets.2019.00023

**Published:** 2019-02-11

**Authors:** Megan R. LaFollette, Kerri E. Rodriguez, Niwako Ogata, Marguerite E. O'Haire

**Affiliations:** ^1^Department of Animal Sciences, College of Agriculture, Purdue University, West Lafayette, IN, United States; ^2^Center for the Human-Animal Bond, Department of Comparative Pathobiology, College of Veterinary Medicine, Purdue University, West Lafayette, IN, United States; ^3^Department of Veterinary Clinical Sciences, College of Veterinary Medicine, Purdue University, West Lafayette, IN, United States

**Keywords:** training methods, human-animal interaction, animal-assisted intervention, service dog, military veterans, PTSD, human-animal bond, IOS

## Abstract

**Introduction:** Psychiatric service dogs are increasingly being sought out by military veterans as a complementary intervention for posttraumatic stress disorder (PTSD). After receiving a service dog, many veterans continue training their service dog at home. Our objective was to explore the associations between training methods, PTSD severity, service dog behavior, and the veteran-service dog bond in a population of military veterans with PTSD.

**Methods:** Post-9/11 military veterans with PTSD who had received a psychiatric service dog were recruited from a national service dog provider. A total of 111 veterans (*M* = 40.1 ± 8.3 years, 80% male) participated in an online survey regarding frequency of training methods, PTSD symptom severity, service dog behavior, and the human-animal bond. Service dogs were predominately Labrador Retriever purebreds or mixes of various breeds (66% male) and mostly obtained from shelters or rescues (58%). Training methods were divided into five categories: positive reinforcement (e.g., physical praise), negative punishment (e.g., ignoring the dog), positive punishment (e.g., verbal correction), dominance (e.g., alpha roll), and bond-based (e.g., co-sleeping). Data were analyzed using general linear models.

**Results:** Veterans self-reported using all five categories of training methods at least once a month. More frequent use of positive punishment was associated with less closeness with their service dog (*p* = 0.02), more fear (*p* = 0.003), less eye contact (*p* < 0.0001), and less trainability (*p* = 0.04). More frequent use of positive reinforcement was associated with higher closeness to their service dog (*p* = 0.002) and perceived increased attachment behavior (*p* = 0.002) and playfulness (*p* = 0.002). More frequent use of bond-based methods was associated with higher closeness to their service dog (*p* = 0.02). PTSD severity was not significantly associated with reported dog behavior, temperament, or veteran-service dog closeness.

**Conclusion:** Military veterans with PTSD service dogs reported using many training methods that were associated with different outcomes. In general, the reported use of positive reinforcement or bond-based training methods were associated with reporting more positive outcomes while the reported use of positive punishment was associated with reporting more negative outcomes. Educating service dog organizations and recipients about the impacts of training methods could be beneficial for service dog efficacy and welfare.

## Introduction

Military veterans with posttraumatic stress disorder (PTSD) are increasingly seeking out complementary therapies such as psychiatric service dogs. PTSD is characterized by intrusion, avoidance, negative alterations in cognition and mood, and alterations in arousal and anxiety (1). PTSD affects an estimated 6–14% of post-9/11 military veterans returning from deployments to Iraq or Afghanistan (2, 3) and is often linked to suicidal behavior (4), major depression (5), and substance abuse (6). Unfortunately, successful treatment of PTSD remains a challenge and current evidence-based treatments for PTSD often have high dropout and non-response rates (7–9). As a complement to evidence-based treatment, many military veterans are seeking out psychiatric service dogs to address their daily PTSD symptoms.

Psychiatric service dogs for PTSD are a specialized type of service dog specifically trained to perform a variety of tasks designed to mitigate the symptoms of PTSD. In the United States, a service dog must be individually trained to do work or perform tasks for a person with a disability ^1^. For individuals with PTSD these tasks may include responding to the veteran's anxiety, “watching” the veteran's back in public, and waking them up from nightmares. If the dog is trained to do this task and is under control of the handler, it is permitted to accompany persons with disabilities in most public places. There are no specific tests required to qualify as a service dog. Regardless of whether a service dog is initially trained by the veteran themselves, a service dog organization, or a third-party trainer, most veterans maintain the service dog's training after placement in the home for optimum application.

Between the organization and the military veteran, a variety of training methods could be used to maintain a service dog's reliability in performing their trained tasks. These training methods could include both specific techniques rooted in operant conditioning theory and specific interactions that may be rooted in a particular style to reinforce a specific relationship with the service dog. Operant conditioning includes four quadrants that can be used in conjunction: positive reinforcement, positive punishment, negative reinforcement, and negative punishment. Positive reinforcement, or reward-based training, is the addition of a rewarding stimuli (i.e., reinforcers) to increase the likelihood of the behavior (i.e., response) occurring again (e.g., giving a dog a treat after it sits). Positive punishment, or aversive-based training, is the addition of an aversive stimuli to decrease the likelihood of the behavior occurring again (e.g., jerking on the leash when a dog pulls). Negative reinforcement is the removal of a punishing or aversive stimulus (i.e., a loud noise or pain) to increase the likelihood of the behavior occurring again (e.g., releasing pressure on the collar when the dog is at your side). Negative punishment is when a rewarding stimuli are removed to decrease the likelihood of the behavior occurring again (e.g., removing attention when a dog jumps). Two additional types of training styles are also present in working dog and service dog organizations: so-called dominance-based (10) and bond-based training (11). Dominance-based training emphasizes the belief that the handler can establish a superior position over the service dog to aid with training (e.g., always eating before a dog or alpha roll). Bond-based training emphasizes the belief that service dogs are best trained by the handler establishing a close bond with their dog (e.g., sharing food with the dog or co-sleeping).

Research suggests that training methods can impact indicators of canine welfare. The use of aversive training methods (e.g., positive punishment) has been found to be related to reduced dog welfare such as stress behaviors during training, elevated cortisol, and problem behaviors such as fear and aggression (12–14). On the contrary, the use of positive reinforcement methods alone has previously been associated with lower dog fear and aggression than other methods (12). Current knowledge on outcomes related to either positive or aversive training methods is limited to companion, police, or laboratory dogs. No previous studies, to our knowledge, have investigated the association between training methods on canine behavior in psychiatric service dogs.

In addition to the effects of training on service dog behavior or welfare, the handler's psychological status may also have an effect on service dogs. For example, a longitudinal study found that owner symptoms of depression and PTSD predicted the development of behavioral problems (aggression, separation anxiety, and attention-seeking behaviors) in search & rescue dogs (15). Additionally, a cross-sectional study found higher aggression in cocker spaniels owned by emotionally unstable owners (16). Finally, a recent study also found a 5-fold increase in the use of aversive training methods in men with moderate depression (17). Currently, the potential relationship between the PTSD symptom severity of military veterans and the behavior of their psychiatric service dogs are unknown. It is important to determine and understand this relationship to enhance the welfare of psychiatric service dogs.

Finally, the human-animal bond between a service dog and handler should be mutually beneficial to both the service dog and the handler (18). For handlers, the human-animal bond has previously been found to be associated with mental, social, and physiological benefits for pet owners (19). For dogs, more strongly bonded pet owners are also most likely to walk their dogs, seek preventative care, and follow health-care recommendations from their veterinarians (20, 21). The bond has previously been shown to be impacted by human attitudes and personality (22), but, to our knowledge, no study has investigated the relationship between training techniques, PTSD severity, and dog behavior on the human-animal bond between military veterans and their service dogs.

The objective of this research was to explore the associations between reported use of training methods, PTSD severity, dog behavior, and the human-animal bond among a population of military veterans and their psychiatric service dogs. Based on previous research, we hypothesized that higher reported use of aversive training methods (i.e., positive punishment or dominance) would be associated with higher perceived negative outcomes (e.g., less closeness, more fear, and more aggression), while higher reported use of positive training methods (i.e., positive reinforcement or bond-based) would be associated with higher perceived positive outcomes (e.g., more closeness, more attention, more trainability). Additionally, we hypothesized that higher PTSD severity would be associated with higher perceived negative outcomes.

## Materials and Methods

The study protocol was approved by the Purdue University Human Research Protection Program Institutional Review Board (IRB Protocol 1607017967). No interactions occurred between the research team and service dogs during the study, therefore we received a waiver from Purdue University's Institutional Animal Care and Use Committee (IACUC).

### Participants

Participants were recruited from K9s For Warriors (Ponte Vedra, Florida, USA), an Assistance Dog International (ADI) accredited, non-profit organization that provides service dogs to military veterans across the United States of America. Participants were military veterans who received a service dog from K9s For Warriors. Our inclusion criteria were (1) military service after September 11, 2001, (2) a community diagnosis of PTSD or meeting the clinical cutoff on the validated PTSD Checklist [PCL; (23)] (3) honorable discharge or current honorable service, (4) no history of or current substance abuse, (5) no conviction of any crime against animals, and (6) no more than two pet dogs currently in the home.

All participants attended a 3-week placement class at K9s For Warriors consisting of a set of standardized training and dog handling instruction. Veterans were instructed to use a combination of reward (e.g., positive reinforcement) and correction (e.g. positive punishment) based training and complete 120 h of training with their service dog over the 3-week period. Training methods were matched to the needs of the individual dog based on assessment from experienced dog trainers. Prior to the class, all dogs had been screened for temperament and trained for at least 60 h using operant conditioning with positive reinforcement and leash corrections. The organization also abides with ADI minimum standards for assistance dogs including training for at least three disability-related tasks, basic obedience skills (i.e., down, recall), and appropriate public behavior (i.e., no signs of aggression, acceptable greeting behaviors, appropriate attention seeking, etc.) (24).

### Procedure

Participants were recruited between January and May of 2016 via an initial email and attached flyer which included detailed information about study participation. Following voluntary informed consent, participation consisted of completing a 10–15 min online survey. Upon completion of this survey, participants chose between receiving $20 in cash or a $20 Amazon gift card in remuneration (55% chose amazon gift card and 45% chose cash). Potential participants received up to 3 follow-up email reminders. Of 244 veterans with a service dog contacted, 111 (45%) participated in the online survey.

### Measures

#### Demographics

Participants were asked to report their age, gender, number of children, number of pet dogs, and the month and year they received their service dog. Participants also consented for the researchers to access their records on file with K9s For Warriors which allowed for the extraction of service dog information including breed, sex, and source (shelter rescue, owner relinquishment, breeder donation, etc.). Dog breed and source were then coded into broad categories to assist with analysis.

#### PTSD Checklist

PTSD symptom severity was assessed using the PTSD Checklist (PCL-5), a widely used 20-item scale based on the four DSM-V symptom clusters of intrusion symptoms (subscale B), avoidance (criterion C), negative alterations in cognitions and mood (criterion D) and alterations in arousal and reactivity [criterion E; (25)]. Participants were asked to indicate the degree to which each PTSD symptom has bothered the participant in the past month on a scale from 0 = not at all to 5 = extremely. A higher PCL score indicated greater overall symptom severity, with a diagnosis cutoff of 31–33 on a scale of 0 to 80 (7, 26).

#### Inclusion of Other in the Self Scale (IOS)

**Figure 1:**
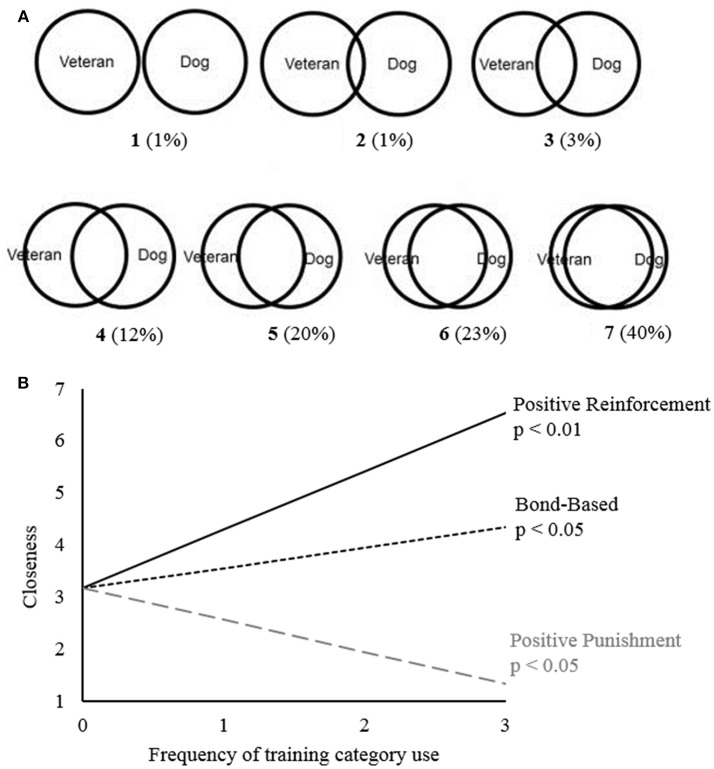
Closeness and Training Methods. Military veterans (*N* = 111) were asked to describe the current relationship between themselves and their service dog (self-perceived closeness) by choosing an item on a pictorial scale. **(A)** The pictorial scale shown to military veterans, replicated from the Inclusion of Others in the Self Scale. In parentheses is the overall percentage of military veterans who chose each picture in this sample. **(B)** Significant associations between reported closeness and training methods resulting from linear regression models. Training Methods Scale: 0 = Never, 1 = Once, 2 = Once a Week, 3 = Daily.

The human-animal bond was assessed with the Inclusion of Other in the Self Scale (IOS), a single question measure that quantifies self-perceived closeness of relationships (27). Participants were asked to describe the current relationship between themselves and their service dog on a pictorial scale (1 = completely separate circles and 7 = highly overlapping circles; Figure 1). The IOS exhibited high reliability in the current sample (Cronbach's α's = 0.93), and has established convergent and divergent validity (27, 28). It correlates well with other interpersonal relationship measures such as the Relationship Closeness Inventory (29), the Subjective Closeness Index (29), the Sterberg Intimacy Scale (30), and the Positive and Negative Emotions about Others scales (27–29).

#### Training Methods

Participants' at-home training methods and frequency of use were evaluated using a questionnaire (Supplemental Table 1) modified from a previous survey of canine training methods (12). In the survey, each training method was described as objectively as possible and given an example such as “Verbal praise (‘good boy').” Participants were asked to estimate how often they had used each training method in the past month (0 = never, 1 = once or twice, 2 = weekly, and 3 = daily). Participants were asked about a wide range of possible training methods (beyond what they were taught by the specific service dog organization) in order to capture their actual in-home interactions with their service dogs. Training methods were grouped into broad categories for analysis based on operant conditioning techniques (positive reinforcement, positive punishment, and negative punishment) and interaction styles (bond-based and dominance-based) commonly used by dog trainers and service dog organizations (Table 1) This survey was pre-tested by canine experts in the field as well as pet dog owners.

**Table 1:** Handler Training Methods.

**Category and behaviors**	**M**	**SD**	**Range**	**% Using daily**
**Positive reinforcement**	**2.2**	**0.3**	**1.2–3**	**100**
Physical praise	3.0	0.2	1–3	100
Verbal praise	2.9	0.5	0–3	96
Play reward	2.5	0.8	0–3	64
Food reward	2.4	0.8	0–3	60
Clicker training	0.1	0.4	0–3	1
**Bond-based**	**1**	**0.6**	**0–3**	**51**
Co sleep	1.8	1.3	0–3	50
“Do as I do”	0.8	1.0	0–3	7
Share Food	0.3	0.6	0–2	0
**Negative punishment**	**0.7**	**0.7**	**0–3**	**10**
Ignore dog	0.7	1.0	0–3	5
Time out	0.6	0.8	0–3	8
**Positive punishment**	**1.6**	**0.6**	**0.3–3**	**79**
Verbal correction	2.7	0.6	1–3	78
Flat collar correction	1.5	1.2	0–3	30
Prong collar correction	1.4	1.1	0–3	23
Physical correction	0.8	1.0	0–3	11
**Dominance-based**	**0.9**	**0.7**	**0–3**	**45**
Eat before	1.4	1.4	0–3	38
Alpha roll	0.7	1.0	0–3	10
Stare down	0.5	0.8	0–3	5

Military veterans (N = 111) self-reported use (mean ± SD, range) of different training behaviors with their psychiatric service dogs.

*Bold indicates the larger category of training method used for analysis as categorized by researchers. Scale: 0 = never, 1 = once or twice, 2 = weekly, 3 = daily (in the last month)*.

Although it is difficult to clearly separate between positive punishment and negative reinforcement, we chose to categorize techniques based on positive punishment and use this term for the remainder of the manuscript. This rationale was 2-fold in that the majority of previous literature focuses on potential negative effects of positive punishment (which may be more salient to the dog) and, second, to simplify the analysis and interpretation by ensuring that each training behavior was only included in a single category.

#### Dog Behavior and Character

Dog behavior and character was evaluated via a modified questionnaire (Supplemental Table 1) from previous surveys of canine behaviors including the Canine Behavioral Assessment and Research Questionnaire (C-BARQ)©(12, 14, 31). Participants were asked to report how often their service dog had displayed a series of behaviors in the last month (0 = never, 1 = rarely, 2 = sometimes, and 3 = often, 4 = always). Each behavior was described as objectively as possible such as “stayed close to you when you're sitting down or resting.” After collecting the data, behaviors were grouped into broad categories for analysis based on previous research studies (12, 14, 31). Dog character was described by directly asking veterans to describe the character of their service dog as best they could on traits such as playfulness, fear, eye contact, and sociability (0 = not at all/never, 1 = a little bit, 2 = moderately/sometimes, 3 = quite a bit, 4 = extremely/always). This survey was pre-tested by canine experts in the field as well as pet dog owners.

### Statistical Methods

Data were analyzed in Statistical Package for the Social Sciences (SPSS 24.0) using a series of regression models. Prior to testing, all assumptions of linear regression were confirmed including the independence of residuals, homogeneity of variance, normality of residuals, and multicollinearity in the data. For all summary scales, an average of individual items was calculated (excluding participants with >50% of missing data in each measure). Data is presented as mean ± standard deviation, where applicable. The significance level was set at *p* < 0.05.

The dependent variable for the veteran-dog bond was the Inclusion of Other in Self (IOS) scale. The dependent variables for service dog behavior were total unwanted behaviors, fear/avoidance, overall aggression, trainability, and attachment-attention behaviors. Finally, the dependent variables for service dog character were each individual item.

In each model, explanatory variables included the frequency of use for each training type or style and the veteran's total PTSD Score. For training methods, the numerical frequency of each method was calculated and then averaged. All statistical models also initially included covariates of veteran age, veteran gender, dog sex, and time since placement. We removed covariates above *p* = 0.10 in the final analyses.

## Results

### Demographics

A total of 111 military veterans participated in the survey. Military veterans were mostly male (80%) with an average age of 40 ± 8 years (range 22–63). Average PCL-5 scores were 44 ± 17 (range 3–80). Veteran-service dog pairs had been together for anywhere from 1 month to 7 years (M = 22 ± 20 months). Service dogs were mostly male (66%) and mostly shelter or rescue dogs (58%) with some from other service dog providers (23%) or other sources (17%; e.g., owner surrender). Their reported breeds were mostly purebred (38%) or mixed (31%) Labrador Retrievers, with a large percentage of other purebred or mixed breeds (31%) such as German Shepherds or Golden Retrievers.

### Training Methods

Veteran service dog handlers self-reported using all five categories of training methods in the past month (Table 1). Positive reinforcement was reported to be used most often with physical (100%; e.g., petting) and verbal praise (96%; e.g., “good boy”) being used by almost all veterans on a daily basis. Positive punishment was the second most commonly used with the majority of veterans using verbal corrections (78%; e.g., “no”) on a daily basis. Bond-based methods were the third most common, which was largely driven by half of veterans (50%) co-sleeping with their dogs daily. Dominance-based methods ranked fourth in frequency which was largely driven by 38% of veterans eating before their service dogs. Finally, negative punishment was used rarely (only 10% using daily).

### PTSD Severity

Veteran's PTSD symptom severity was not significantly associated with any service dog behaviors, service dog character, or the veteran-dog bond (all *p*'s > 0.05, Table 2).

**Table 2:** Associations between training methods, PTSD Severity, veteran-service dog closeness (IOS), and service dog behavior and character.

	**Positive reinforcement**	**Bond-based**	**Negative punishment**	**Positive punishment**	**Dominance- based**	**PTSD severity**	**Gender**	**Age**	**Time**
**HUMAN-ANIMAL BOND**									
IOS	0.294^**^	0.211^*^	0.057	−0.249^*^	0.000	0.067	−0.317^**^		
**SERVICE DOG BEHAVIOR**									
All Problems	−0.060	0.053	0.194	0.160	0.079	0.039	0.207^*^	0.305^**^	
Trainability	0.234^*^	0.017	−0.188	−0.230^*^	0.087	0.011		−0.199^*^	
Attachment/Attention	0.307^**^	0.053	0.076	−0.225	0.096	0.044			
Fear/Avoidance	−0.029	0.161	0.228	0.175	−0.036	0.000	0.172	**0.282**	
Aggression	−0.070	−0.018	0.128	0.070	0.075	0.032		0.181	
**SERVICE DOG CHARACTER**									
Playfulness	0.316^**^	−0.175	0.186	−0.094	−0.169	−0.028		−0.241^*^	
Activity	0.293^**^	−0.232^**^	0.138	−0.123	−0.082	−0.074			
Fear	−0.129	0.088	0.064	0.344^**^	−0.118	0.082			
Eye contact	0.168	0.182	0.148	−0.419^***^	0.002	0.016			
Chase drive	0.275^**^	−0.128	0.252^*^	−0.216	−0.171	−0.047			−0.190
Focus	0.192	−0.032	−0.122	−0.028	0.034	0.162		0.234^*^	
Sociability	0.035	0.162	0.088	0.231	−0.115	−0.007			0.198
Reactivity	0.067	−0.005	0.195	−0.073	−0.182	0.092			
Food drive	0.121	−0.006	−0.168	0.028	0.045	−0.038			

The associations (standardized regression coefficients, β) from linear regression models of self-report data from military veterans (N = 111) about their training method usage, PTSD severity, human-animal bond, service dog behavior, and service dog character. Gender reference category was male. Time was the number of months since the veteran had received his or her dog. Blank cells indicate that the covariate was not included in the final model, with p > 0.10. Bold indicates a significant effect.

* p < 0.05,

** p < 0.01,

*** *p < 0.001*.

### Closeness

Military veterans felt extremely close to their service dogs (M = 5.8 ± 1.3, maximum of 7, Figure 1). More frequent reported use of positive reinforcement and bond-based methods were associated with a closer bond; conversely, more frequent reported use of positive punishment was associated with a less close bond (Table 2, Figure 1). Additionally, male military veterans reported a closer bond to their service dogs than female veterans.

### Service Dog Behavior and Character

#### Behavior

Participants reported that their service dogs often exhibited behaviors often interpreted as indicative of attachment or trainability and more rarely exhibited negative behaviors (such as those indicative of aggression or fear; Table 3). For example, over half of veterans reported that their service dog *always* follows them from room to room when at home (68%), stays close when sitting down or resting (60%), obeys a sit (66%), and listens closely to them (61%). Anxiety and fear behaviors were reported next frequently with over 40% of service dogs reported to show behaviors of anxiety or fear at least *sometimes*. For example, 46% of service dogs were reported to at least *sometimes* be anxious or upset when alone. Finally, behaviors potentially indicative of aggression were reported least often. However, 16% of service dogs displayed at least one potentially aggressive behavior *often* or *always* in the past month, with 10% of service dogs displaying unwanted barking at the veteran *often* or *always*.

**Table 3:** Service dog behavior.

**Category and behaviors**	***M***	***SD***	**Range**	**% Often or always**
**Attachment or attention-seeking**	**3.2**	**0.6**	**1–4**	**97**
Solicits attention	3.5	0.7	1–4	91
Follows from room to room	3.5	0.8	1–4	90
Stays close by	2.9	1.0	0–4	75
Makes & holds eye contact	2.6	1.1	0–4	55
**Trainability**	**3.3**	**0.5**	**2–4**	**100**
Obeys “sit”	3.6	0.5	1–4	100
Listens closely	3.5	0.6	1–4	96
Obeys “stay”	3.5	0.7	0–4	92
Comes immediately when called	3.0	1.1	0–4	75
Distracted^*^	1.7	0.9	0–4	15
Steals food^*^	0.4	0.7	0–4	2
**Anxiety & fear**	**1**	**0.7**	**0–3**	**37**
Hid, shook, or paced from loud noises	1.5	1.4	0–4	25
Anxious or upset when alone	1.0	1.3	0–4	18
Anxious or upset when in public	0.9	1.0	0–4	9
Cautious or shy around new people	0.4	0.7	0–4	1
**Potential aggression**	**0.4**	**0.5**	**0–2**	**16**
Nipping at veteran	0.5	0.9	0–4	7
Nipping at other people	0.2	0.5	0–3	1
Nipping at other dogs	0.3	0.7	0–4	3
Unwanted growling at veteran	0.6	1.0	0–4	7
Unwanted growling at other people	0.4	0.9	0–4	5
Unwanted growling at other dogs	0.0	0.2	0–1	0
Unwanted barking at veteran	0.7	1.1	0–4	10
Unwanted barking at other people	0.6	0.9	0–4	7
Unwanted barking at other dogs	0.1	0.4	0–3	1

Military veterans reported the frequency that their psychiatric service dog (N = 111) performed each behavior in the last month. Bold indicates the larger category used for analysis as coded by the researchers.

* *indicates that the item was reverse coded for final analysis. Scale: 0 = never, 1 = rarely, 2 = sometimes, 3 = often, 4 = always*.

In this study, no particular training method was associated with total behavior problems, overall aggression, or behaviors indicative of anxiety and fear in dogs (Table 2). However, certain training methods were associated with attachment or attention seeking behaviors and trainability (Table 2). Specifically, more frequent use of positive reinforcement was associated with increased trainability as well as attachment and attention-seeking behaviors; conversely, more frequent use of positive punishment was associated with less trainability. Factors such as veteran age and gender were also associated with service dog behaviors. Younger veterans reported fewer total behavior problems, fewer fearful and avoidance behaviors, and greater trainability. Male veterans reported fewer total problematic behaviors in dogs.

#### Character

On a scale from 0 to 4, most veterans described their service dogs character as extremely food driven (3.3 ± 1), focused (3.4 ± 1), making eye contact frequently (3.2 ± 1), sociable (3.0 ± 1), playful (2.9 ± 1), and active (2.9 ± 1). Dogs were reported to be quite a bit chase driven (2.5 ± 1) and reactive (2.5 ± 1). Although on average dogs were reported to rarely be fearful (1.1 + 1), 31% of veterans described their dogs as at least moderately fearful in new areas or with new objects.

Certain training techniques and styles were associated with aspects of service dog character (Table 2). Veterans that reported using more positive reinforcement described their dogs as being more playful, having more activity, and being more chase driven. Additionally, more frequent reported use of bond-based methods was associated with lower activity. Conversely, more frequent reported use of positive punishment was associated with higher fear and less eye contact. Additionally, more frequent reported use of negative punishment was associated with higher chase drive. Finally, younger veterans reported higher playfulness and greater focus in their service dogs. Neither the use of certain training technique or styles nor any covariates were associated with food drive, reactivity, or sociability (all *p*'s > 0.05).

## Discussion

### General

To our knowledge, this study represents the first to compare associations between reported use of different training methods, PTSD severity, the veteran-dog bond, and dog behavior or character among military veterans with PTSD and their service dogs. Our results did not support our hypothesis that veteran PTSD severity would be associated with negative outcomes, but provided mixed evidence of other our hypotheses.

Our results provided mixed evidence in support of our first hypothesis that self-reported aversive training methods would be significantly associated with negative outcomes. Specifically, veterans who reported more frequent use of positive punishment reported less closeness with their service dog and perceiving their service dogs as exhibiting more fear, less eye contact, and being less trainable. However, there was no association between positive punishment and aggression (discussed below) or dominance-based training methods and any outcomes.

Our results also provided mixed evidence in support of our second hypothesis that self-reported positive training methods would be significantly associated with positive outcomes. Specifically, veterans who reported more frequent positive reinforcement reported more closeness, attention, trainability, and playfulness with their service dog. Veterans who reported more frequent bond-based training reported more closeness with their service dogs.

### Training Methods

Military veterans in the population surveyed used a wide variety of in-home training methods with their psychiatric service dogs. Since we only asked veterans to report what training methods they were currently using (and did not ask them to specify the reasons they chose their methods) it is likely that these methods are based not only on instruction from the service dog organization, but also previous experience training dogs or seeing others training dogs such as military working dogs or through television programs. All veterans used some amount of positive reinforcement daily (e.g., physical praise, food rewards) and almost all veterans used some positive punishment daily (e.g., verbal correction, leash correction), which aligned with the service dog organization's instruction and recommendations. In comparison, bond-based, dominance-based, and negative punishment training methods were used less often. In terms of bond-based techniques, 50% of veterans reported sleeping in the same bed as their dog, which may be partially due to the fact that some dogs are trained to wake their veterans up during nightmares.

A comprehensive review of previous studies indicates that aversive training methods (e.g., positive punishment and dominance-based training) have been correlated with indicators of compromised welfare in dogs such as stress-related behaviors during training, impaired human-dog bond, elevated cortisol, and problem behaviors such as fear and aggression (13). However, this review also notes that many of the previous studies were non-objective surveys focused mainly on police and laboratory dogs, which may not be representative of the larger dog population and do not indicate causal direction. That is, with a correlational study—as in our current study—it is impossible to know whether behavior problems were caused by aversive methods or increased used of aversive methods were caused by behavior problems (or even if the two are not causally related, but just associated). Furthermore, the previous objective empirical studies have mainly focused on using shock-collars in training (13), which were never used in our population.

In terms of positive reinforcement, there have been perhaps even fewer formal investigations of its impact on indicators of dog welfare. One observational study did show that dogs from a school using positive-reinforcement showed increased attentiveness toward their owner, while dogs from a school using negative-reinforcement showed signals of stress (32). Reward-based training has also been found to correlate with obedience (14, 33). However, in one of these studies increased use of reward-based training was also associated with increased owner-reported canine aggression and excitability (33), which seems to be contrary to other findings.

There have been even fewer formal investigations of dominance-based training methods (although several discussions of the concept), bond-based training, and negative punishment. For dominance, a survey of dog owners of dogs with behavior problems, directly confrontational methods (including dominance and positive punishment methods such as alpha roll, stare down, physical correction) were reported to elicit an aggressive response from dogs and therefore not recommended (34). Furthermore, scientific reviews on using dominance as a construct in domestic dogs agree that using coercive methods to assert “dominance” (i.e., alpha roll) is counterproductive, unsafe for owners, likely to negatively impact dog welfare, and is associated with undesirable behaviors (35–37). In this study, a lack of findings for dominance-based training methods could be because we included the behavior of “eating before” the dog (based off of common practices in dominance-based training books), which can simply provide structure and routine for the dog and is unlikely to be particularly aversive. Additionally, some veterans may not actually perform “alpha rolls” in an aversive manner to establish dominance. In the survey, we attempted to describe this behavior as objectively as possible [“force dog to roll on their back (‘alpha roll')”] to prevent response bias, but in doing so lost the context of the actions. Therefore, it is possible that some veterans perform this behavior in a more playful manner that may not actually be aversive to the dogs.

For bond-based training (although no specific techniques have been assessed) there has been an association that owners who allow their dogs to sleep in their bedroom have higher attachment to their animals (38). Overall, it is clear that scientific evidence is limited in determining the effect of dog training techniques on dog welfare, training efficacy, and the human-animal bond. Our study took an initial approach to evaluating the associations between training methods, dog behavior, and the human-animal bond among military veterans and their psychiatric service dogs.

### Closeness

Overall, veterans reported high interrelationship closeness with their service dogs, with 40% of veterans choosing the highest degree of circular overlap between themselves and their dogs, and the mean for all veterans being 5.8 on a 7-point scale. The Inclusion of Other in the Self-Scale (IOS) is a fairly novel measure in the human-animal bond literature; it indicated that veterans both feel close and perform behaviors associated with closeness with their service dogs (27). Our results align fairly well with previous results that handlers of service dogs have higher closeness with their dogs than pet owners. Previously, using the IOS, pet owners have been found to have a mean of 3.5 and 3.9 out of 7 with their closest pet (39, 40), while inmates training service dog puppies were found to have higher means of 6.2 (41). The IOS is advantageous because it is a single item scale that is fast for participants to complete and is not reliant on participants having a specific type of bond, but instead relies on individual perceptions. It also appears to not have the ceiling effect previously seen in other service dog owners (42).

There was no significant association detected between severity of PTSD symptomology and veteran-service dog closeness. This suggests that regardless of the severity of PTSD experienced, veterans are still able to bond strongly with their service dogs. This is mirrored by findings that there is no association between the Monash Dog Owner Relationship Scale and PTSD symptoms among military veterans (O'Haire and Rodriguez, Unpublished data).

There were a few associations between veteran-service dog closeness and self-reported use of training techniques. In particular, we found that both positive reinforcement and bond-based training techniques were associated with closer bonds. Positive reinforcement techniques include verbal praise and bond-based methods including co-sleeping may increase perceived closeness (22, 43). Conversely, we found that greater use of positive punishment was associated with less closeness. However, as this is an association-based study, we cannot determine causality. For example, it is possible that veterans who feel less close to their dogs are more likely to use positive punishment, rather than the use of positive punishment causing less close feelings.

### Service Dog Behavior and Character

There was no significant relationship observed between veteran-reported service dog behavior or character and PTSD symptom severity. This suggests that veterans with more severe PTSD may not cause or perceive behavioral problems in their service dogs. This is contrary to previous results finding that emotional instability and symptoms of depression and PTSD are associated with and predicted the development of behavioral problems in pet and search-and-rescue dogs (15, 16). Therefore, it is possible that this result may be unique to specifically trained PTSD service dogs.

Overall, service dogs displayed many positive behaviors and character. Most service dogs frequently showed behaviors typically interpreted as signs of trainability as well as attachment & attention behaviors. This is unsurprising as service dogs are specifically selected and trained to be highly attentive and obedient to their handlers. Their character was generally appropriate for a service dog with most dogs being highly food driven and displaying frequent eye contact. A high display of eye contact is important because of literature showing that eye contact increases the production of oxytocin in both dogs and humans and facilitates owners' affiliative behaviors (44). Increasing oxytocin production is particularly relevant to veterans with PTSD as the application of intranasal oxytocin has been suggested as a complementary strategy for PTSD treatment (45).

The most common problem behavior category cited by veterans was signs of fear and anxiety. Veterans reported that 45% of their service dogs were at least sometimes anxious or upset when left alone. Previously, signs of owner-reported separation anxiety of pet dogs has been measured at rates between ~34–38% (12, 14). Service dogs are very rarely left alone since they are allowed to accompany their handlers in public places. Therefore, this issue may be less observable in service dogs than pet dogs simply because it occurs less often. However, because service dogs are rarely left alone, it may leave dogs less prepared to be alone when they must be, which will undoubtedly occur occasionally. Relatively high levels of separation anxiety may also relate to service dog training to form high attachment with their owners–as indicated by most service dogs always following their owners around at home–which is also considered as a potential signal of separation anxiety. Signs of at least rare fear of noises were reported in 46% of service dogs which is similar to previous studies of companion dogs where percentages range from 12.1 to 43% (12, 46, 47). On the contrary, 94% of service dogs in our study never or rarely showed signs of anxiety when in public, which is important since public access is the main feature distinguishing a service dog from a pet dog.

Although there was no association between overall behavior problems in dogs and training techniques, there were several associations between behavior and character subscales and training techniques. Positive methods such as positive reinforcement and bond-based training generally were associated with more positive behaviors such as higher eye contact, attachment and attention behaviors, and playfulness. These findings support prior research that positive reinforcement was associated with lower undesirable behaviors (12, 14). On the contrary, positive punishment was associated with more signs of fear, less eye contact, and less trainability. This finding supports previous work indicating associations and causality of negative outcomes when positive punishment is used (12–14). However, it is possible that handlers who find their dogs less trainable are more likely to use positive punishment, rather than positive punishment causing less trainability. However, this alternative explanation makes less sense when considering the association between self-reported fear and positive punishment; that is, it less logical for handlers who perceive their dogs are more fearful to use positive punishment to combat that, rather than positive punishment actually leading to higher fear. Finally, increased use of negative punishment was slightly associated with higher perceived chase drive toward balls or moving objects. It is possible that dog's that chase more frequently are also subject to techniques such as “time outs” in the crate, rather than negative punishment actually causing increased chasing.

Some veterans reported potentially aggressive behaviors occurring–albeit at very low levels and rates–such as unwanted barking or growling at other people. The American Disabilities Act requires that service dogs must be under control of the handler at all times ^2^; however, these results do not necessarily indicate that the dogs are not under control or even showing true aggression. We did not distinguish as to whether these instances occurred in public situations or while the dog was in the home. Furthermore, in the comment section of this section of the survey, some veterans noted that the dog aggression was toward off-leash dogs that had approached the service dog while working or mouthy-ness during normal play with the family dog. Additionally, some unwanted barking could be due to excitement or attention seeking behavior. However, other veterans noted in the comment section of the survey that some growling was due to dogs becoming protective of their handlers.

Other than training methods, there were a few factors that were also associated with service dog behavior and character. Younger veterans reported that their dogs had fewer negative behaviors (both overall and specifically anxiety/fear) and more positive behaviors (playfulness and trainability). It is possible that younger veterans may simply be more able to prevent negative behaviors and elicit positive behaviors or be more effective dog trainers. On the other hand, they simply may have a more positive view of their service dogs and report fewer problems and more positive behaviors.

### Limitations

There are several limitations to this investigation. First, since this study was cross-sectional it is impossible to determine causation in the associations that were uncovered. For example, it is possible that veterans who feel closer to their dogs are simply more likely to use positive reinforcement techniques, rather than positive reinforcement actually causing more feelings of closeness. Further studies would benefit from randomly assigning training methods to subsets of the population to determine the direction of causality of this association. However, this study provides initial insight into associations between training methods and relevant outcomes, which could provide rationale for future study.

Second, this survey only evaluated veterans receiving service dogs from a single service dog provider. This may have reduced possible variation in our results and masked additional relationships that could be identified. However, as we did find acceptable variation and this is one of the largest providers of PTSD service dogs that serves a nationally representative sample of veterans, the results may still be applicable to a wide population.

Finally, since this survey only included indirect, handler-reported behavioral assessments of their service dogs, there is the potential for subjective biases to occur. Additionally, handlers reported behaviors may not accurately reflect their training styles for the best assessment of dominance- or bond-based training styles. Further studies would benefit from objective behavioral observations with either live or video coding, assessment of the context of these behaviors, and an assessment of the handlers' overall training philosophy. However, this study provides insight into the experiences and perceptions of veterans with service dogs, which are uniquely important to consider in the context of an intervention targeting human-perceived outcomes. Additionally, handler perceptions of dog behavior are critical to understand as they likely influence the human-animal bond, which is the basis for the practice of service dogs for PTSD.

## Conclusions

In conclusion, there appear to be associations between higher reported use of positive training methods and positive outcomes for service dogs. Additionally, there are a few associations between higher reported use of negative training methods and negative outcomes for service dogs. Finally, there was no association between PTSD severity, closeness between a veteran and their service dog, or the dog's behaviors or character. Overall, educating service dog organizations and recipients about the relationships between training methods, service dog behavior, and service dog character could be beneficial for service dog efficacy and welfare.

## Author Contributions

All authors contributed to the conceptualization and methodology of the study. ML and KR contributed to data curation. ML, KR, and MO performed the statistical analysis. ML wrote the first draft of the manuscript. All authors contributed to manuscript revision, read and approved the submitted version.

### Conflict of Interest Statement

The authors declare that the research was conducted in the absence of any commercial or financial relationships that could be construed as a potential conflict of interest.

## Footnotes

^1^Americans With Disabilities Act of 1990, Pub. L. No. 101-336, 104 Stat. 328. (1990).

^2^Americans With Disabilities Act of 1990. (1990). 104.

## Supplementary Material

Supplemental Table 1 Questionnaire for military veterans. The question scale, question text, response options, and coded response values of the survey given to military veterans about their PTSD service dogs.
